# SGLT inhibitor adjunct therapy in type 1 diabetes

**DOI:** 10.1007/s00125-018-4671-6

**Published:** 2018-08-22

**Authors:** Rory J. McCrimmon, Robert R. Henry

**Affiliations:** 1 School of Medicine, Mail Box 12, Level 7 University of Dundee, Ninewells Hospital and Medical School, Dundee, DD1 9SY UK; 2 Division of Endocrinology and Metabolism, School of Medicine, UC San Diego, La Jolla, CA USA

**Keywords:** Clinical trials, Diabetic ketoacidosis, GLP-1 receptor agonist, HbA_1c_, Hypoglycaemia, Insulin, Review, Sodium–glucose cotransporter inhibitors, Type 1 diabetes, Weight

## Abstract

Non-insulin adjunct therapies in type 1 diabetes have been proposed as a means of improving glycaemic control and reducing risk of hypoglycaemia. Evidence to support this approach is, however, scant and few pharmacological agents have proved effective enough to become part of routine clinical care. Recent short-term Phase II trials and 24 week Phase III trials provide initial support for the use of sodium–glucose cotransporter (SGLT) inhibitors in type 1 diabetes. Two international, multicentre, randomised, controlled clinical trials, Dapagliflozin Evaluation in Patients with Inadequately Controlled Type 1 Diabetes (DEPICT-1) and inTandem3, have reported that SGLT inhibition with dapagliflozin and sotagliflozin, respectively, confer additional benefits in terms of a 5–6 mmol/mol (0.4–0.5%) reduction in HbA_1c_ accompanied by weight loss and reductions in total daily insulin doses. The reduction in HbA_1c_ does not come with a significantly increased risk of hypoglycaemia but does carry an increased risk of diabetic ketoacidosis and mycotic infections. These results suggest that SGLT inhibition will have a place in the management of type 1 diabetes. Longer-term clinical trials (≥52 weeks) and observational cohort studies are needed to determine any additional benefits or adverse effects of this adjunct therapy and to determine which group of patients may benefit most from this approach. In addition, use of SGLT inhibitors in routine type 1 diabetes care will require specific patient and healthcare professional educational packages to ensure patient safety and to minimise risk.

## Introduction

Chronic exposure to hyperglycaemia in type 1 diabetes carries an increased risk of microvascular [1] and macrovascular disease [2]. Intensive insulin therapy aimed at optimal glucose control can largely prevent or minimise these complications, especially when combined with appropriate blood pressure control and lipid-lowering therapy [1, 2]. Current insulin delivery systems are limited by peripheral drug delivery and lack of feedback inhibition. This, together with the impaired response to hypoglycaemia in type 1 diabetes, means that intensive insulin therapy is associated with increased glucose variability, weight gain and severe hypoglycaemia [3–5]. Hence, most individuals with type 1 diabetes do not achieve recommended glycaemic targets [6, 7] and overall life expectancy is still substantially lower than in the non-diabetic population [8]. Meeting the twin challenges of hyperglycaemia and hypoglycaemia in type 1 diabetes may require additional adjunct pharmacotherapies to complement insulin replacement [9].

## Adjunct non-insulin pharmacological interventions in type 1 diabetes

Initial trials of adjunct therapy in type 1 diabetes were small, short-term and generally had unimpressive outcomes [9]. Pramlintide, currently licensed only in the USA, was one of few agents shown to be effective, but its beneficial effects on HbA_1c_ and weight were small and the risk of hypoglycaemia was increased [10]. Of the dipeptidyl peptidase-4 inhibitors, sitagliptin is the most widely studied as adjunct therapy in type 1 diabetes, but no major benefit was seen in three 52 week RCTs [11–13]. More recently, two large multicentre RCTs, ADJUNCT ONE (*N* = 1400) [14] and REMOVAL (REducing with MetfOrmin Vascular Adverse Lesions; *N* = 428) [15], examined the effectiveness of the glucagon-like peptide-1 receptor agonist liraglutide (1.2 mg and 1.8 mg doses) and metformin, respectively, as adjuncts to insulin therapy in type 1 diabetes. In ADJUNCT ONE, liraglutide showed significant benefits over placebo in terms of HbA_1c_, weight and total insulin dose reduction. However, the impact on HbA_1c_ was not impressive (mean 0.15–0.2% reduction) and the rates of symptomatic hypoglycaemia and hyperglycaemia with ketosis increased significantly. Accordingly, Novo Nordisk, the sponsor of the ADJUNCT trials, did not pursue a label indication for liraglutide in type 1 diabetes. Similarly, despite the frequent off-label use of metformin in type 1 diabetes [9, 16], in the REMOVAL study it failed to significantly reduce carotid intima–media thickness (the primary endpoint) and early small reductions in HbA_1c_ and body weight were not sustained [15].

In summary, while the rationale for adjunct therapy in type 1 diabetes is clear, there is little robust evidence to support the adjunctive use of currently available therapies.

## Phase II clinical trials of SGLT inhibition in type 1 diabetes

Sodium–glucose cotransporters (SGLTs) are found predominantly in the mucosa of the small intestine (SGLT1) and the proximal tubules of the kidney (SGLT2 and SGLT1) [13]. SGLT2 inhibition reduces glucose reabsorption in the renal tubule, leading to increased glucose excretion. SGLT1 inhibition reduces dietary glucose and galactose absorption in the intestine and augments the release of gastrointestinal incretins. Selective SGLT2 inhibitors (empagliflozin, dapagliflozin, canagliflozin) or dual SGLT1/SGLT2 inhibitors (sotagliflozin) are therefore an attractive therapeutic proposition for diabetes because increased urinary glucose excretion will reduce hyperglycaemia and, through loss of energy (each gram of glucose lost is equivalent to 16.7 kJ [4 cal]), facilitate weight loss [17]. While there is an obvious place for such a therapy in type 2 diabetes, pre-clinical research in rodent models also suggest that these agents may be beneficial as an adjunct therapy in type 1 diabetes (e.g. [18, 19]).

In an open-label, proof-of-concept trial in 40 young adults with long-duration type 1 diabetes, Perkins et al reported on the effects of empagliflozin 25 mg once daily for 8 weeks. Participants were asked to reduce prandial and basal insulin by 30% on initiation of treatment [20], with further dose adjustments being made based on capillary glucose measures. Empagliflozin co-therapy reduced HbA_1c_ by 4 ± 5 mmol/mol (0.3 ± 0.4%; mean ± SD) and total daily insulin requirements were decreased, largely as a result of a reduction in basal insulin (from 25.7 ± 10.6 to 19.5 ± 7.9 U/day, *p* < 0.0001) [20]. Mean body weight also decreased (from 72.6 ± 12.7 to 70.0 ± 12.3 kg, *p* < 0.0001) and, despite the fall in HbA_1c_, symptomatic hypoglycaemia <3.0 mmol/l and all hypoglycaemia <3.9 mmol/l measured using continuous glucose monitoring (CGM) were less frequent compared with baseline. Two participants withdrew early because of diabetic ketoacidosis (DKA) (associated with severe gastroenteritis in one and pump failure in the other).

In a placebo-controlled, double-blind, parallel-group study using empagliflozin 2.5 mg, 10 mg or 25 mg daily with insulin for 4 weeks, Pieber et al [21] found benefits in terms of HbA_1c_ (4–5 mmol/mol [0.4%] reduction), total insulin dose (0.07–0.09 U/kg reduction) and weight (1.5–1.9 kg reduction) (all *p* < 0.05). Similarly, Henry et al randomly assigned 70 adults with type 1 diabetes (HbA_1c_ 53–86 mmol/mol [7–10%]), stabilised on insulin, to receive dapagliflozin (1, 2.5, 5 or 10 mg) or placebo over 2 weeks [22]. Insulin doses were not proactively reduced but were adjusted based on capillary blood glucose for safety. Dapagliflozin increased glucosuria dose-dependently, with the highest dose (10 mg) resulting in a urinary glucose excretion of 88 g/24 h (95% CI 55, 121) [22]. This dose reduced 24 h average glucose (−2.29 mmol/l [95% CI −3.71, −0.87]) and mean amplitude of glycaemic excursion (−3.77 mmol/l [95% CI −6.09, −1.45]), both assessed by CGM. Total daily insulin dose was reduced by 16.2% (95% CI 0.5, 29.4) [22].

An 18 week, double-blind, Phase II randomised study involving 315 adults with long-duration type 1 diabetes assessed the effect of canagliflozin 100 mg or 300 mg daily vs placebo as adjunct therapy [23]. Participants initially down-titrated insulin doses by 10–20% (based on baseline HbA_1c_), followed by dose adjustments according to capillary blood glucose. Significant improvements in the following variables were produced by canagliflozin 100 mg and 300 mg, respectively (placebo-subtracted least mean squares differences from baseline): HbA_1c_ (mean change −3.2 mmol/mol [−0.3%] and − 2.7 mmol/mol [−0.3%]); body weight (mean change −3.4% and −5.3%) and total insulin dose (absolute mean change in total insulin dose −4.1 U/day [−8.9%] and −7.6 U/day [−12.9%]). The latter was largely driven by reductions in basal insulin. In this trial, DKA was experienced by 5.1% and 9.4% of the participants receiving canagliflozin 100 mg and 300 mg, respectively, and by none in the placebo arm. Rates of hypoglycaemia were broadly similar across all groups, although more episodes of severe hypoglycaemia occurred with canagliflozin 300 mg [23]. Based on these findings, further development of the programme for canagliflozin in type 1 diabetes was stopped.

Finally, in a 4 week randomised, placebo-controlled, double-blind trial in 33 adults with long-duration type 1 diabetes, Sands et al [24] studied the effect of the dual SGLT1/2 inhibitor sotagliflozin. Compared with placebo, HbA_1c_ decreased by 5.3 mmol/mol (0.5%) (*p* ≤ 0.01) from baseline, as did total insulin daily dose (approximately 15%; *p* < 0.05). In contrast to selective SGLT2 inhibitors, this dual inhibitor was primarily associated with reduced bolus insulin (−26%, *p* < 0.01). Post-meal AUC for glucose and mean amplitude of glycaemic excursion were also significantly reduced, as was body weight (−1.7 kg vs −0.5 kg, *p* < 0.01). There was no increase in the rate of hypoglycaemia, although two participants experienced DKA, possibly due to pump failure [24].

## Phase III clinical trials with SGLT inhibitors in type 1 diabetes

The first two Phase III trials of SGLT inhibitors in type 1 diabetes were published in September 2017. In inTandem3 [25], a 24 week double-blind, placebo-controlled RCT, 1402 people with type 1 diabetes were randomised to receive either sotagliflozin (400 mg/day) or placebo after a 2 week single-blind run-in period (Table 1). Based on lessons learned from the Phase II studies, participants were instructed to reduce meal-time insulin by 30% with the first dose of study drug and then subsequently adjust insulin based on capillary blood glucose. Participants received information on the detection and treatment of DKA and were provided with blood ketone meters. The cohorts were well-matched at baseline and were mostly (88%) white adults (aged 42 ± 14 years) with long-duration type 1 diabetes (20 ± 12 years) and an average baseline HbA_1c_ of 66 ± 10 mmol/mol (8.2 ± 0.9%) [21]. Sixty per cent of the participants were on multi-dose insulin (MDI) and 40% were on insulin-pump therapy. The pre-specified composite endpoint (HbA_1c_ <53 mmol/mol [7.0%] at week 24, with no episodes of severe hypoglycaemia or DKA) was achieved in significantly more participants taking sotagliflozin than placebo (28.6% vs 15.2% [95% CI 9.0, 17.8], *p* < 0.001) (Table 2). In the whole-group analysis, from baseline to week 24 there was a greater change in HbA_1c_ with sotagliflozin (difference −6 mmol/mol [−0.5%], *p* < 0.001) plus a greater reduction in body weight (difference −2.98 kg, *p* < 0.001) and a reduction in placebo-corrected alterations in the mean daily total, bolus and basal doses of insulin (difference −5.3 U/day [−9.7%], −2.8 U/day [−12.3%] and −2.6 U/day [−9.9%], respectively, *p* < 0.001 for all comparisons). In participants with a baseline systolic blood pressure (SBP) of >130 mmHg, the reduction in SBP by week 16 was greater with sotagliflozin (difference −3.5 mmHg, *p* = 0.002 vs placebo) (Table 2). Sotagliflozin 400 mg daily was relatively well tolerated compared with placebo (overall rate of any adverse event was 52–55%), although serious adverse events were more common with sotagliflozin (48 participants [6.9%] vs 23 [3.3%]) (Table 3). Documented hypoglycaemia and hypoglycaemia event rates were similar in both groups, although sotagliflozin-treated participants had a significantly lower event rate of hypoglycaemia <3.1 mmol/l. As expected, general mycotic infections and diarrhoea occurred more frequently with sotagliflozin vs placebo and a greater proportion of participants experienced one or more episodes of DKA (3.0% vs 0.6%) [21].

**Table 1:** Summary of trial design, duration, primary and secondary endpoints and participant baseline characteristics in currently available literature on Phase III studies involving SGLT inhibitor adjunct therapy in type 1 diabetes

Study detail	Dapagliflozin	Sotagliflozin		
Study name	DEPICT-1	InTandem1	InTandem2	InTandem3
Design	Optimise diabetes management	Optimise insulin	Optimise insulin	Standard of care insulin
Study arms	Three (PBO, DAPA 5 mg, DAPA 10 mg)	Three (PBO, SOTA 200 mg, SOTA 400 mg)	As for inTandem1	Two (PBO, SOTA 400 mg)
Total *N*	833	793	782	1402
Study duration	52 weeks^a^	52 weeks^a^	52 weeks^a^	24 weeks
Primary endpoint	HbA_1c_ change from baseline at week 24	Reduction in HbA_1c_ vs PBO on optimised insulin (24 weeks)	As for inTandem1	Proportion with HbA_1c_ <53 mmol/mol (<7.0%) no SH and no DKA (24 weeks)
Secondary key endpoints	Proportion with HbA_1c_ decrease of ≥6 mmol/mol (≥0.5%) without SH at 24 weeks; % change in total daily insulin; % change in body weight; CGM change in mean 24 h glucose, MAG, % 24 h readings within target range	Proportion with HbA_1c_ <53 mmol/mol (<7.0%), no SH, and no DKA; body weight, bolus insulin dose, FPG, DTSQ, DDS2	As for inTandem1	HbA_1c_, body weight, SBP, bolus insulin dose
Mean age of participants	42 years	46 years	41 years	42 years
Mean duration of type 1 diabetes	20 years	24 years	18 years	20 years
Method of insulin delivery	40% CSII; 60% MDI	60% CSII; 40% MDI	26% CSII; 74% MDI	40% CSII; 60% MDI
Mean BMI at baseline	28 kg/m^2^	30 kg/m^2^	28 kg/m^2^	28 kg/m^2^
Mean HbA_1c_ at screening	73 mmol/mol (8.8%)	66 mmol/mol (8.2%)	68 mmol/mol (8.4%)	68 mmol/mol (8.4%)
Mean HbA_1c_ at baseline	69 mmol/mol (8.5%; after 8 weeks’ lead-in)	60 mmol/mol (7.6%; after 6 weeks’ optimisation)	61 mmol/mol (7.7%; after 6 weeks’ optimisation)	66 mmol/mol (8.2%)
Mean baseline SBP	Not reported	120 mmHg	123 mmHg	122 mmHg
eGFR inclusion criteria	Not reported	≥45 ml min^−1^ [1.73 m]^−2^	≥45 ml min^−1^ [1.73 m]^−2^	≥45 ml min^−1^ [1.73 m]^−2^

These are not head-to-head studies

^a^This table shows 24 week data from DEPICT, inTandem1 and inTandem2

CSII, Continuous subcutaneous insulin infusion (pump); DAPA, dapagliflozin; DDS2, Diabetes Distress Scale 2; DTSQ, Diabetes Treatment Satisfaction Questionnaire; FPG, fasting plasma glucose; MAG, mean absolute glucose change; PBO, placebo; SH, severe hypoglycaemia; SOTA, sotagliflozin

**Table 2:** Summary of major placebo-adjusted efficacy measures from reported Phase III studies involving SGLT inhibitor adjunct therapy in type 1 diabetes

Efficacy measure (PBO adjusted)	Dapagliflozin	Sotagliflozin		
	DEPICT-1^a^	inTandem1^a^	inTandem2^a^	inTandem3
HbA_1c_ change	DAPA 5 mg: −4.6 mmol/mol (−0.42%) (*p* < 0.0001) DAPA 10 mg: −5.0 mmol/mol (−0.45%) (*p* < 0.0001)	SOTA 200 mg: −4.0 mmol/mol (−0.36%) (*p* < 0.001) SOTA 400 mg: −4.5 mmol/mol (−0.41%) (*p* < 0.001)	SOTA 200 mg: −4.0 mmol/mol (−0.36%) (*p* < 0.001) SOTA 400 mg: −3.9 mmol/mol (−0.35%) (*p* < 0.001)	SOTA 400 mg: −5.1 mmol/mol (−0.46%) (*p* < 0.001)
Secondary (composite) endpoint and per cent achieving this	HbA_1c_ reduction ≥6 mmol/mol (≥0.5%) without SH PBO: 25% DAPA 5 mg: 50% DAPA 10 mg: 51%	HbA_1c_ <53 mmol/mol (<7.0%), no SH, no DKA PBO: 22% SOTA 200 mg: 34% SOTA 400 mg: 44%	HbA_1c_ <53 mmol/mol (<7.0%), no SH, no DKA PBO: 15% SOTA 200 mg: 31% SOTA 400 mg: 32%	HbA_1c_ <53 mmol/mol (<7.0%), no SH, no DKA PBO: 15.2% SOTA 400 mg: 28.6% HbA_1c_ reduction ≥6 mmol/mol (≥0.5%) without SH PBO: 36.7% SOTA 400 mg: 60.9%
Change from baseline in total insulin dose (%)	DAPA 5 mg: −8.8% (*p* < 0.0001) DAPA 10 mg: −13.2% (*p* < 0.0001)	NA	NA	SOTA 400 mg: −9.71% (*p* < 0.001)
Mean change in SBP	NA	SOTA 200 mg: −5.4 mmHg (*p* = 0.017) SOTA 400 mg: −6.6 mmHg (*p* = 0.003)	NA	SOTA 400 mg: −3.5 mmHg (*p* = 0.002)
Mean change from baseline in body weight	DAPA 5 mg: −2.96% (*p* < 0.0001) DAPA 10 mg: −3.72% (*p* < 0.0001)	SOTA 200 mg: −2.4 kg (*p* < 0.001) SOTA 400 mg: −3.5 kg (*p* < 0.001)	SOTA 200 mg: −2.0 kg (*p* < 0.001) SOTA 400 mg: −2.6 kg (*p* < 0.001)	SOTA 400 mg: −3.0 kg (*p* < 0.001)

^a^This table shows 24 week data from DEPICT, inTandem1 and inTandem2

DAPA, dapagliflozin; NA, data not publicly available; PBO, placebo; SH, severe hypoglycaemia; SOTA, sotagliflozin

**Table 3:** Number of major adverse outcomes of interest reported in published Phase III studies involving SGLT inhibitor adjunct therapy in type 1 diabetes

Adverse event of special interest	Dapagliflozin	Sotagliflozin		
	DEPICT-1^a^	InTandem1^a^	InTandem2^a^	InTandem3
Genital infection	PBO: 7 (3%) DAPA 5 mg: 34 (12%) DAPA 10 mg: 33 (11%)	PBO 8 (3.0%) SOTA 200 mg: 16 (6.1%) SOTA 400 mg: 27 (10.3%)	PBO 4 (1.6%) SOTA 200 mg: 19 (7.3%) SOTA 400 mg: 20 (7.6%)	PBO: 15 (2.1%) SOTA 400 mg: 45 (6.4%)
Diarrhoea	Not reported	PBO: 14 (5.2%) SOTA 200 mg: 17 (6.5%) SOTA 400 mg: 26 (9.9%)	PBO: 8 (3.1%) SOTA 200 mg: 10 (3.8%) SOTA 400 mg: 15 (5.7%)	PBO: 16 (2.3%) SOTA 400 mg: 29 (4.1%)
Serious adverse events^b^	PBO: 15 (6%) DAPA 5 mg: 19 (7%) DAPA 10 mg: 24 (8%)	PBO: 3.4% SOTA 200 mg: 3.8% SOTA 400 mg: 6.9%	PBO: 3.5% SOTA 200 mg: 4.2% SOTA 400 mg: 4.2%	PBO: 23 (3.3%) SOTA 400 mg: 48 (6.9%)
Deaths^b^	PBO: 1 (<1%) DAPA 5 mg: 0 DAPA 10 mg: 0	PBO 0 SOTA 200 mg: 0 SOTA 400 mg: 0	PBO 2 SOTA 200 mg: 0 SOTA 400 mg: 0	PBO: 2 SOTA 400 mg: 1
Severe hypoglycaemia	PBO: 19 (7%) DAPA 5 mg: 21 (8%) DAPA 10 mg: 16 (6%)	PBO: 18 (7%) SOTA 200 mg: 11 (4%) SOTA 400 mg: 12 (5%)	PBO: 7 (3%) SOTA 200 mg: 10 (4%) SOTA 400 mg: 6 (2%)	PBO: 17 (2%) SOTA 400 mg: 21 (3%)
DKA	PBO: 3 (1%) DAPA 5 mg: 4 (2%) DAPA 10 mg: 5 (2%)	PBO: 0 (0%) SOTA 200 mg 3 (1%) SOTA 400 mg: 8 (3%)	PBO: 0 (0%) SOTA 200 mg: 2 (1%) SOTA 400 mg: 4 (2%)	PBO: 4 (1%) SOTA 400 mg: 21 (3%)
DKA on CSII	Not reported	PBO: 0 (0%) SOTA 200 mg: 2 (1%) SOTA 400 mg: 6 (4%)	PBO: 0 (0%) SOTA 200 mg: 0 (0%) SOTA 400 mg: 3 (5%)	PBO: 2 (1%) SOTA 400 mg: 12 (4%)
DKA on MDI	Not reported	PBO: 0 (0%) SOTA 200 mg: 1 (1%) SOTA 400 mg: 2 (2%)	PBO: 0 (0%) SOTA 200 mg: 2 (1%) SOTA 400 mg: 1 (1%)	PBO: 2 (1%) SOTA 400 mg: 9 (3%)

^a^This table shows 24 week data from DEPICT, inTandem1 and inTandem2

^b^Where values are not presented as *n* (%), these data were not available

CSII, continuous subcutaneous insulin infusion (pump); DAPA, dapagliflozin; PBO, placebo; SH, severe hypoglycaemia; SOTA, sotagliflozin

The 24 week data from two further ongoing clinical trials (inTandem1 and inTandem2) have been published as abstracts [26, 27] (Tables 1–3). Placebo-adjusted effects of sotagliflozin 200 mg and 400 mg in these two RCTs after 24 weeks were similar to those reported in inTandem3.

The second Phase III trial was the Dapagliflozin Evaluation in Patients with Inadequately Controlled Type 1 Diabetes (DEPICT-1) trial [28], a double-blind, parallel-controlled, three-arm, 24 week study in 833 individuals with type 1 diabetes, in which participants were randomised to receive dapagliflozin 5 mg or 10 mg or placebo (Table 1) after a run-in period of 8 weeks to optimise glycaemic control. Participants were asked to reduce both basal and bolus insulin by up to 20% on the day of study drug initiation and to adjust subsequent doses based on self-monitoring of blood glucose four to six times daily. Two periods (each lasting 2 weeks) of blinded CGM were also included. Participants received education on DKA and were provided with blood ketone meters. As in inTandem3, most participants were white, with a mean age of 42.5 (±13.9) years and a duration of type 1 diabetes of 20.3 (±11.8) years (Table 1) [28]. In this trial, the addition of dapagliflozin (5 mg or 10 mg) vs placebo to type 1 diabetes therapy resulted in a significant reduction HbA_1c_ (mean change from baseline at week 24–5 mmol/mol [−0·42%] [95% CI −0·56, −0·28] and −4 mmol/mol [−0·45%] [95% CI −0·58, −0·31] for dapagliflozin 5 mg and 10 mg, respectively, both *p* < 0.0001 vs placebo) (Table 2). This improvement in HbA_1c_ was accompanied by significant reductions in body weight (mean change at week 24 was −2.96% [95% CI −3.63, −2.28] and −3.72% [95% CI −4.38, −3.05] for dapagliflozin 5 and 10 mg, respectively, both *p* < 0.001 vs placebo) and total daily insulin dose (mean difference −8.8% [95% CI −12.6, −4.9] and −13.2% [95% CI −16.8, −9.4] for dapagliflozin 5 mg and 10 mg, respectively, *p* < 0.001 vs placebo). The proportional reductions seen for basal and bolus insulin doses individually were similar in percentage to the total insulin dose reduction for each of the dapagliflozin doses. CGM revealed modest but significant reductions in glucose variability with both doses of dapagliflozin. For instance, the time spent in the target glucose range (>3.9 mmol/l to <10.0 mmol/l) was increased from 43.2 ± 12.4% to 52.3 ± 14.8% (*p* < 0.05) after 24 weeks of dapagliflozin 10 mg. Again, adverse events were not uncommon, with more genital infections occurring with dapagliflozin vs placebo (Table 3). Hypoglycaemia (all categories) did not occur more frequently with dapagliflozin. DKA was infrequent in all groups (1–2%) and was not increased significantly by dapagliflozin [28]. However, adjudication of suspected DKA differed between the DEPICT-1 and inTandem3 trials and rates of DKA would have been similar had both trials adopted the same criteria (2.5% increase with treatment vs placebo groups) [29].

## Summary

Most people with type 1 diabetes do not achieve recommended glycaemic targets. Adjunct therapy may complement insulin replacement and enable more people to achieve their glycaemic goals but there has been limited evidence to support this approach. Two recent RCTs, inTandem3 and DEPICT-1, suggest that SGLT inhibition may prove to be a viable and effective adjunct therapy in type 1 diabetes [25, 28]. Considering the Phase II and III trials together, on average, addition of SGLT inhibitors to insulin replacement in type 1 diabetes resulted in a 5–6 mmol/mol (0.4–0.5%) reduction in HbA_1c_, a 3–4 kg weight loss and a 10–15% reduction in total daily insulin dose. The glucose-lowering effect of SGLT inhibitors is insulin independent and glucose dependent and is accompanied by reduced glucose variability. Hypoglycaemia rates are not increased by SGLT inhibition but there is an associated increased risk of DKA.

DKA seems to occur more frequently in pump-treated patients; the use of rapid-acting insulin alone in pumps means there is no basal insulin back-up as in MDI treatment. An elegant study by Patel et al [30] suggests that the increased risk of DKA is predominantly due to the failure of type 1 diabetes patients on SGLTi to promptly recognise early metabolic decompensation, which occurs at lower than usual glucose levels, rather than being due to any acceleration in the rate of ketogenesis following the interruption of basal insulin infusion. Most healthcare organisations advocate blood ketone testing if capillary glucose is >16.7 mmol/l (<300 mg/dl) or persistently >13 mmol/l (235 mg/dl) and an individual is feeling unwell. However, capillary glucose may not rise above 11–12 mmol/l (200–215 mg/dl) despite significant ketosis in individuals with type 1 diabetes receiving SGLT inhibitor treatment [30]. Education of patients concerning the risk of DKA at lower than expected glucose levels is therefore crucial, together with advice on sick-day rules and routine provision of a blood ketone meter (see Text box). Clinicians should consider whether it is safe to prescribe SGLT2 inhibitors for individuals who are poorly compliant, whose blood glucose is poorly controlled (HbA_1c_ >75 mmol/mol [9%]) and who are most at risk for hospitalisation from DKA [31]. It may also be advisable to limit SGLT inhibition to those on MDI rather than insulin pump, unless steps can be taken to minimise the risk of pump failure, which would result in abrupt discontinuation of insulin. In addition, using the lowest available dose of SGLT inhibitor may reduce the risks of DKA and other adverse events. Education of patients should include revision of insulin:carbohydrate ratios for calculating bolus insulin, perhaps by accounting for predicted glucose loss in the urine, and to ensure total insulin dose (especially basal) is not reduced by more than 10–15%.

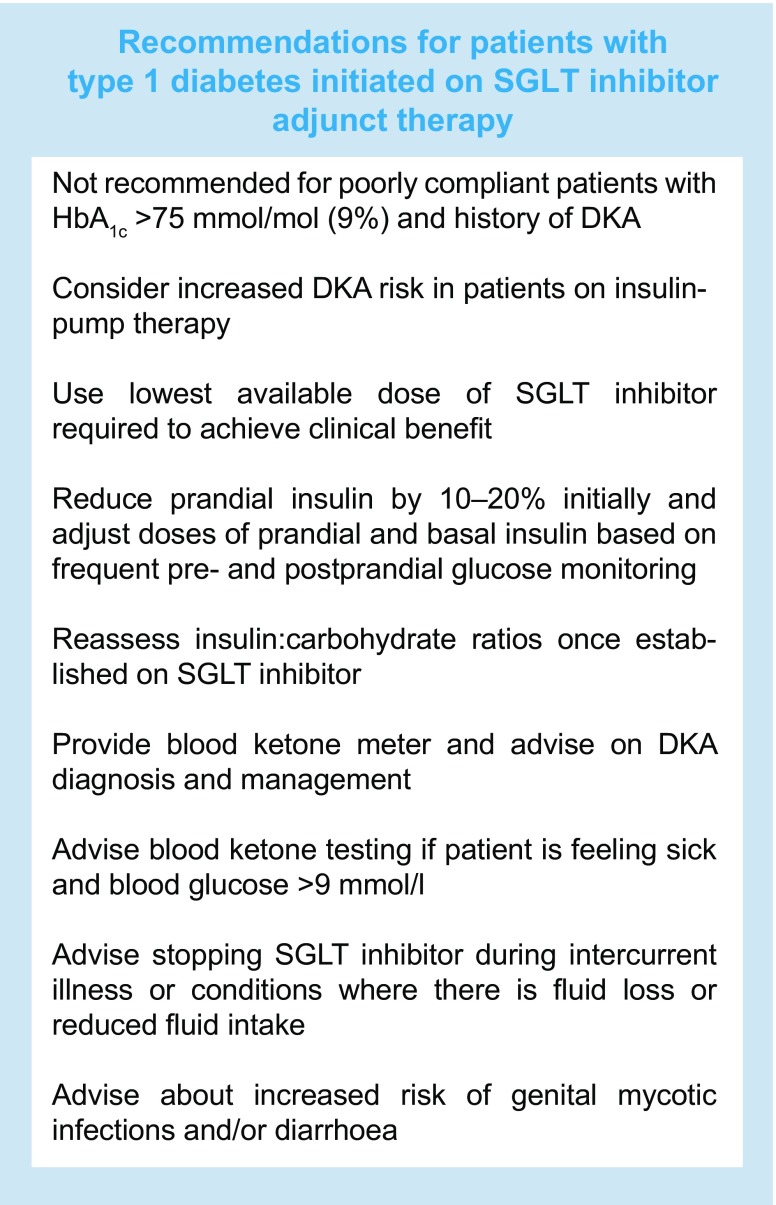



It remains to be clarified whether dual SGLT1/2 inhibition, which in the short-term appears to have a greater effect on postprandial glucose control, confers additional benefits in terms of HbA_1c_ or other outcomes over the longer term. Upcoming results from 52 week RCT trials (DEPICT-2, inTandem1, inTandem2, Empagliflozin as Adjunctive to inSulin thErapy Over 52 Weeks in Patients with Type 1 Diabetes Mellitus [EASE-2]) will help to determine whether SGLT inhibitor adjunct therapy has sustained benefits in terms of HbA_1c_, weight and total insulin dose reduction and determine its impact on rates of DKA and severe hypoglycaemia. Data on long-term clinical efficacy, safety (especially less-common adverse events), survival, health-related quality of life, patient-reported outcomes and resource requirements will require longer-term observational cohort studies. Future trials might determine whether the beneficial effects of SGLT inhibition on blood pressure [25] and arterial stiffness [32] translate into improved cardiovascular outcomes (still a major cause of increased morbidity and mortality in type 1 diabetes [8]), whether SGLT inhibitors have a reno-protective effect in type 1 diabetes (as in type 2 diabetes [33]) and does dual SGLT1/2 inhibition have additional benefits in those with impaired renal function because of the reduction in gastrointestinal glucose uptake. Alternatively, will early animal data showing these compounds can increase renal excretion of calcium [34], and results from CANVAS indicating higher fractures rates in participants with type 2 diabetes taking canagliflozin [35], translate into an already increased risk of bone fractures in type 1 diabetes?

Taken together, early data are promising but further research is needed to define clearly the cohort of people with type 1 diabetes who will benefit most from SGLT inhibitor co-prescription as well as to obtain more data on safety and persistence of benefit in the longer term. SGLT inhibitors are not currently approved by the US Food and Drug Administration or the European Medicines Agency for use in type 1 diabetes but they may become a therapeutic option in the future. Education of patients and healthcare professionals will be of paramount importance if SGLT inhibitors are to be introduced safely into routine clinical care of type 1 diabetes.

## Abbreviations

CGM: Continuous glucose monitoring
DEPICT-1: Dapagliflozin Evaluation in Patients with Inadequately Controlled Type 1 Diabetes
DKA: Diabetic ketoacidosis
MDI: Multi-dose insulin
REMOVAL: REducing with MetfOrmin Vascular Adverse Lesions
SBP: Systolic blood pressure
SGLT: Sodium–glucose cotransporter

## References

[1] Nathan DM, Genuth S, Diabetes Control and Complications Trial Research Group (1993). The effect of intensive treatment of diabetes on the development and progression of long-term complications in insulin-dependent diabetes mellitus. N Engl J Med.

[2] Nathan DM, Cleary PA, Backlund JY (2005). Intensive diabetes treatment and cardiovascular disease in patients with type 1 diabetes. N Engl J Med.

[3] Beall C, Ashford ML, McCrimmon RJ (2012). The physiology and pathophysiology of the neural control of the counterregulatory response. Am J Phys Regul Integr Comp Phys.

[4] Diabetes Control and Complications Trial Research Group (1997). Hypoglycemia in the Diabetes Control and Complications Trial. Diabetes.

[5] The DCCT Research Group (1988). Weight gain associated with intensive therapy in the diabetes control and complications trial. Diabetes Care.

[6] McKnight JA, Wild SH, Lamb MJ (2015). Glycaemic control of type 1 diabetes in clinical practice early in the 21st century: an international comparison. Diabet Med.

[7] Miller KM, Foster NC, Beck RW (2015). Current state of type 1 diabetes treatment in the U.S.: updated data from the T1D Exchange clinic registry. Diabetes Care.

[8] Livingstone SJ, Levin D, Looker HC (2015). Estimated life expectancy in a Scottish cohort with type 1 diabetes, 2008-2010. JAMA.

[9] George P, McCrimmon RJ (2013). Potential role of non-insulin adjunct therapy in type 1 diabetes. Diabet Med.

[10] Edelman S, Garg S, Frias J (2006). A double-blind, placebo-controlled trial assessing pramlintide treatment in the setting of intensive insulin therapy in type 1 diabetes. Diabetes Care.

[11] Griffin KJ, Thompson PA, Gottschalk M, Kyllo JH, Rabinovitch A (2014). Combination therapy with sitagliptin and lansoprazole in patients with recent-onset type 1 diabetes (REPAIR-T1D): 12-month results of a multicentre, randomised, placebo-controlled, phase 2 trial. Lancet Diabetes Endocrinol.

[12] Hari Kumar KV, Shaikh A, Prusty P (2013). Addition of exenatide or sitagliptin to insulin in new onset type 1 diabetes: a randomized, open label study. Diabetes Res Clin Pract.

[13] Zhao Y, Yang L, Xiang Y (2014). Dipeptidyl peptidase 4 inhibitor sitagliptin maintains β-cell function in patients with recent-onset latent autoimmune diabetes in adults: one year prospective study. J Clin Endocrinol Metab.

[14] Mathieu C, Zinman B, Hemmingsson JU (2016). Efficacy and safety of liraglutide added to insulin treatment in type 1 diabetes: the ADJUNCT ONE treat-to-target randomized trial. Diabetes Care.

[15] Petrie JR, Chaturvedi N, Ford I (2017). Cardiovascular and metabolic effects of metformin in patients with type 1 diabetes (REMOVAL): a double-blind, randomised, placebo-controlled trial. Lancet Diabetes Endocrinol.

[16] Vella S, Buetow L, Royle P, Livingstone S, Colhoun HM, Petrie JR (2010). The use of metformin in type 1 diabetes: a systematic review of efficacy. Diabetologia.

[17] Ferrannini E, Solini A (2012). SGLT2 inhibition in diabetes mellitus: rationale and clinical prospects. Nat Rev Endocrinol.

[18] Luippold G, Klein T, Mark M, Grempler R (2012). Empagliflozin, a novel potent and selective SGLT-2 inhibitor, improves glycaemic control alone and in combination with insulin in streptozotocin-induced diabetic rats, a model of type 1 diabetes mellitus. Diabetes Obes Metab.

[19] Powell DR, Doree D, Jeter-Jones S, Ding ZM, Zambrowicz B, Sands A (2015). Sotagliflozin improves glycemic control in nonobese diabetes-prone mice with type 1 diabetes. Diabetes Metab Syndr Obes.

[20] Perkins BA, Cherney DZ, Partridge H (2014). Sodium-glucose cotransporter 2 inhibition and glycemic control in type 1 diabetes: results of an 8-week open-label proof-of-concept trial. Diabetes Care.

[21] Pieber TR, Famulla S, Eilbracht J (2015). Empagliflozin as adjunct to insulin in patients with type 1 diabetes: a 4-week, randomized, placebo-controlled trial (EASE-1). Diabetes Obes Metab.

[22] Henry RR, Rosenstock J, Edelman S (2015). Exploring the potential of the SGLT2 inhibitor dapagliflozin in type 1 diabetes: a randomized, double-blind, placebo-controlled pilot study. Diabetes Care.

[23] Henry RR, Thakkar P, Tong C, Polidori D, Alba M (2015). Efficacy and safety of canagliflozin, a sodium-glucose cotransporter 2 inhibitor, as add-on to insulin in patients with type 1 diabetes. Diabetes Care.

[24] Sands AT, Zambrowicz BP, Rosenstock J (2015). Sotagliflozin, a dual SGLT1 and SGLT2 inhibitor, as adjunct therapy to insulin in type 1 diabetes. Diabetes Care.

[25] Garg SK, Henry RR, Banks P (2017). Effects of sotagliflozin added to insulin in patients with type 1 diabetes. N Engl J Med.

[26] Buse JB, Garg SK, Rosenstock J, Banks P, Sawhney S, Strumph P (2017). Twenty-four week efficacy and safety of sotagliflozin, a dual SGLT1 and SGLT2 inhibitor, as adjunct therapy to insulin in type 1 diabetes (inTandem 1). Diabetes.

[27] Danne T, Cariou B, Banks P, Sawhney S, Strumph P (2017). 24-week efficacy and safety of sotagliflozin, a dual SGLT1 and SGLT2 inhibitor, as adjunct therapy to insulin in type 1 diabetes (inTandem2). Diabetologia.

[28] Dandona P, Mathieu C, Phillip M (2017). Efficacy and safety of dapagliflozin in patients with inadequately controlled type 1 diabetes (DEPICT-1): 24 week results from a multicentre, double-blind, phase 3, randomised controlled trial. Lancet Diabetes Endocrinol.

[29] Garg SK, Strumph P (2018). Effects of sotagliflozin added to insulin in type 1 diabetes. N Engl J Med.

[30] Patel NS, Van Name MA, Cengiz E (2017). Altered patterns of early metabolic decompensation in type 1 diabetes during treatment with a SGLT2 inhibitor: an insulin pump suspension study. Diabetes Technol Ther.

[31] Govan L, Maietti E, Torsney B (2012). The effect of deprivation and HbA_1c_ on admission to hospital for diabetic ketoacidosis in type 1 diabetes. Diabetologia.

[32] Cherney DZ, Perkins BA, Soleymanlou N (2014). The effect of empagliflozin on arterial stiffness and heart rate variability in subjects with uncomplicated type 1 diabetes mellitus. Cardiovasc Diabetol.

[33] Livingstone SJ, Looker HC, Hothersall EJ (2012). Risk of cardiovascular disease and total mortality in adults with type 1 diabetes: Scottish registry linkage study. PLoS Med.

[34] Thrailkill KM, Nyman JS, Bunn RC (2017). The impact of SGLT2 inhibitors, compared with insulin, on diabetic bone disease in a mouse model of type 1 diabetes. Bone.

[35] Neal B, Perkovic V, Mahaffey KW (2017). Canagliflozin and cardiovascular and renal events in type 2 diabetes. N Engl J Med.

